# Low prevalence of symptomatic thyroid diseases and thyroid cancers in HIV-infected patients

**DOI:** 10.1038/s41598-019-56032-7

**Published:** 2019-12-19

**Authors:** Martina Properzi, Tommaso della Giustina, Sara Mentasti, Francesco Castelli, Annacarla Chiesa, Natalia Gregori, Eugenia Quiros-Roldan

**Affiliations:** 1 Department of Infectious and Tropical Diseases, University of Brescia and ASST Spedali Civili Hospital, Brescia, Italy; 2 Department of Medical and Surgical Specialties, Radiological Sciences and Public Health, Unit of Hygiene, Epidemiology and Public Health, University of Brescia, Brescia, Italy

**Keywords:** HIV infections, Thyroid diseases

## Abstract

Thyroid diseases (TDs) have been widely associated with HIV infection. However, data about TDs prevalence and distribution are controversial, and few published studies are available. The aim of our study was to assess prevalence and risk factors of symptomatic thyroid disturbances, including thyroid cancers, in a large cohort of HIV-infected patients. A retrospective cohort study was performed at the Department of Infectious and Tropical Diseases of the University of Brescia, Italy, in the period 2005–2017. We identified all HIV-positive patients with a diagnosis of symptomatic TD in the electronic database of our Department (HIVeDB); we also operated a record-linkage between our data and the Health Protection Agency database (HPADB) of Brescia Province. Multivariate logistic regression analysis was used to determine risk factors associated with TDs onset; an incidence rate analysis was also performed. During the study period, 6343 HIV-infected patients have been followed at our Department; 123 received a diagnosis of symptomatic TD (1.94% of the entire cohort). In the TDs group, almost half of patients were females (n = 59, 48%), mean age was 47.15 years (SD: 11.56). At TD diagnosis, mean T CD4+ cell count was 491 cell/uL and most patients showed undetectable HIV-RNA (n = 117, 95.12%). Among them, 81 patients were found to have hypothyroidism (63 with Hashimoto’s thyroiditis), 21 hyperthyroidism (17 suffered from Graves’ disease), while 11 subjects were diagnosed with a primitive thyroid cancer. Papillary thyroid cancer was the most frequent histotype (n = 7, 63.63%), followed by medullary (n = 2, 18.18%) and follicular thyroid cancer (n = 1, 9.1%). Male gender was a protective factor for TDs development, especially for hypothyroidism (p < 0.001); age emerged as a variable associated with both hypothyroidism (p = 0.03) and thyroid cancer (p = 0.03), while CD4+ cell nadir <200 cell/mm^3^ was associated with symptomatic hyperthyroidism (p = 0.005). To conclude, symptomatic thyroid dysfunctions rate in well-treated HIV-infected patients is low. Age and gender are crucial elements in the onset of thyroid abnormalities, together with T CD4+ cell nadir. Interestingly, medullary thyroid cancer seems to be much more frequent in HIV-infected patients compared to the general population.

## Introduction

Various alterations in endocrine homeostasis were described in HIV-infected patients^1–3^. In particular, thyroid diseases (TDs) have been widely associated with HIV infection since the start of the epidemic, long before the beginning of the combination antiretroviral therapy (cART) era^4–6^.

However, there are controversial data concerning thyroid dysfunctions in people living with HIV (PLWH), as previous studies reported both a higher than expected prevalence of TDs^7–13^ and no apparent increased prevalence of TDs in this population^14–17^. In well-treated HIV-positive individuals, subclinical hypothyroidism^7,9,11–13,18^ and isolated low free thyroxine (FT4) levels^6,10,19^ are the most common described alterations. Anyway, although it is estimated that around one third of PLWH may present biochemical alterations of thyroid function, only 1–3% develops an overt illness^9^.

Many factors could be implicated in the pathogenesis of thyroid disturbances in PLWH. Firstly, HIV itself seems to play a crucial role, determining different thyroid alterations over the course of the disease^5,6^. Furthermore, several opportunistic infections as from Cytomegalovirus (CMV), *Mycobacterium avium*, *Cryptococcus neoformans*, *Pneumocystis jirovecii* (previously known as *P. carinii*), *Aspergillus spp, Mycobacterium tuberculosis* as well as AIDS-defining neoplasms (Kaposi’s Sarcoma, lymphomas) can occasionally involve thyroid gland, rarely resulting in thyroid dysfunction^6,20^. Antiretroviral therapy can also be implicated in TDs. Stavudine has been linked to lower FT4 levels and hypothyroidism^7,8,11,19^, whereas the role of other regimens, as integrase (INIs) and protease inhibitors (PIs), in impairing thyroid function has yet to be fully investigated. Other drugs, as phenytoin, carbamazepine and lithium, that are frequently used among PLWH for psychiatric disorders, can also modify thyroid hormone levels.

Moreover, the improvement of host immune response induced by cART could trigger autoimmune disorders, leading to the appearance of aberrant thyroid autoantibodies. Thus, late after the introduction of antiretroviral therapy, approximately 2% of PLWH develops Graves’ disease^14,21,22^.

Lastly, Hepatitis C virus (HCV) coinfection is frequently observed in PLWH, due to similar transmission routes. HCV has been associated with different autoimmune conditions, including thyroid disorders, although the actual causative mechanisms have not been entirely determined^23,24^. Furthermore, interferon therapy can also promote autoimmune reactions in HCV-infected patients, especially targeting thyroid gland^25^.

To date, few published papers regarding thyroid dysfunctions in HIV-positive patients are available and thyroid alterations prevalence and distribution in PLWH still remain controversial, since previous studies show sample, definitions and outcomes heterogeneity. Moreover, to our knowledge, no studies focusing only on symptomatic thyroid dysfunctions have been performed and data about thyroid primitive tumors in HIV-infected patients are completely lacking.

Thus, the aims of this study were to assess the prevalence of symptomatic thyroid dysfunctions, including primitive thyroid cancers, in a large cohort of HIV-infected patients in the modern cART era and to evaluate their laboratory and clinical characteristics. Finally, we also determined the risk factors associated with the occurrence of symptomatic thyroid abnormalities in this cohort.

## Methods

### Setting and study population

We performed a retrospective cohort study including all HIV-infected patients aged more than 18 years old and receiving cART in charge at the Department of Infectious and Tropical Diseases, ASST Spedali Civili and University of Brescia, Italy. The period of follow-up ranged from January 2005 to December 2017.

We considered as symptomatic TDs all thyroid disorders needing medical or surgical treatment and all thyroid primitive tumors. Symptomatic thyroid dysfunctions were subsequently classified in three groups as follows: hypothyroidisms, hyperthyroidisms and thyroid cancers (TCs).

We identified all HIV-positive patients with a symptomatic TD in the electronic database used for clinical management at our Department (HIVeDB); we also performed a record-linkage between HIV-infected patients followed by our Department and the Health Protection Agency database (HPADB) of Brescia Province, which tracks all services provided by the National Health Service. In this way, we captured all subjects with HIV infection who received in-hospital services and medical treatment for hypo-/hyperthyroidism or had a medical certificate with an ICD9-CM code for TD (including thyroid cancers). Uncertain TD cases were confirmed with phone-call to patients.

Patients’ demographic, epidemiological, clinical and laboratory (biochemical and viro-immunological) data were recorded in an electronic file. Information about coinfections, eventual comorbidities and medication history (including cART) were also collected. In particular, the following parameters were recorded at TD diagnosis and at the last available visit performed at our Clinic: HIV-RNA (cut-off of 37 copies/ml) and lymphocyte T CD4+ and CD8+ cell counts.

The outcomes of the research were TDs, considered in general and as specified diagnosis-related groups (hypothyroidism, hyperthyroidism and thyroid tumors). They were chosen arbitrarily *a priori*, considering scientific evidence and convenience to retrieve data.

### Statistical analysis

Continuous variables were reported as mean with standard deviation (±SD), categorical variables as frequency with percentages. We used chi-squared tests and Fisher exact tests for dichotomous variables comparison and t-test or Wilcoxon rank-sum test for group means comparison (for normal and non-normal distributions, respectively).

Odds ratios (ORs) were computed through multivariate logistic regression. Our model computed ORs for all outcomes, considering CD4+ nadir (as a dichotomous variable with cut-off: 200 cell/mm^3^), gender, age at HIV test (per 10-years older) and HCV coinfection. All the OR estimates were reported together with their 95% confidence intervals (95% CIs) and the p value.

We performed the incidence rate analysis for TDs (considered all together) using periods of three years, from 2005 to 2016. Analysis were stratified by gender. Subjects with less than 30 days of follow up were excluded from the incidence analysis but kept in all the others. We excluded prevalent cases. For this analysis we did not include incident cases during 2017 for getting homogeneous periods.

Moreover, two sensitivity analyses excluding patients with positive HCV antibodies and patients with an AIDS-defining diagnosis were conducted for both the incidence and the prevalence of TDs.

All statistical tests were two-sided at the level of significance p < 0.05. Analyses were performed using Stata software version 14.0 (StataCorp, College Station, TX, USA).

### Ethics

This study was conducted according to the Declaration of Helsinki and the principles of Good Clinical Practice (GCP). As this study had a retrospective design and was based on routinely collected data, patients’ informed consent was not required according to the Italian law (Italian Guidelines for classification and conduction of observational studies, established by the Italian Drug Agency, “Agenzia Italiana del Farmaco – AIFA” on March 20, 2008). Moreover, for this study we used the general authorization of the Italian Guarantor for the use of retrospective demographical and clinical data, which have been treated according to present laws.

## Results

### Characteristics of study cohort

From 2005 to 2017, 6343 HIV-infected patients have been followed at our Department with at least one visit; 71.68% was represented by males, mean age at the end of follow-up was 47.30 ± 11.90 years. HCV/HIV coinfection was present in 39.08% of the cohort. A total of 123 patients showed a symptomatic TD in our HIVeDB during the study period (1.94% of the entire cohort including prevalent and incident cases). All detected subjects were confirmed by the HPADB and no additional cases were identified using this source.

### Characteristics of patients with TD (n = 123)

**Figure 1:**
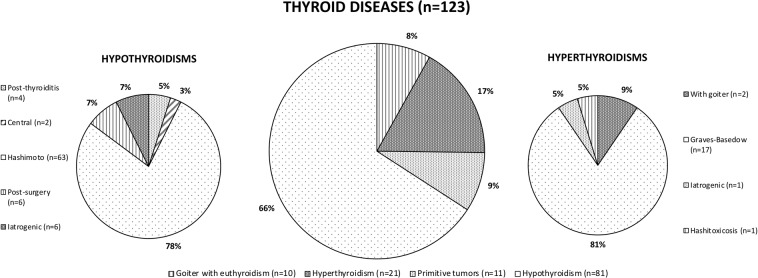
Distribution of symptomatic thyroid diseases (TDs) in the cohort considered.

All patients were known to be HIV-infected when they developed thyroid dysfunction; mean age at TD diagnosis was 46.6 years (SD: 11.5). Characteristics of the study population at baseline and at the end of follow-up are summarized in Table 1. In the TDs group, almost half of patients were females (n = 59, 48%), 67 subjects (54.5%) showed a CD4+ lymphocytes nadir <200/mm^3^ and 71.54% (n = 88) experienced a previous AIDS-defining event. Mean time from HIV to TD diagnosis was 11.04 years (SD: 9.60); at TD diagnosis, mean T CD4+ cell count was 491 cell/uL (range 1–1556 cell/uL) and most patients had undetectable HIV-RNA (n = 117, 95.12%). The logistic regression analysis showed that TDs were negatively associated with male gender (OR = 0.52, 95% CI 0.35‐0.77; p < 0.001) and positively associated with age at HIV test per 10-year increase (OR = 1.24, 95% CI 1.06‐1.45; p = 0.005). At the end of follow-up, all patients with TD had serum thyroid stimulating hormone (TSH), serum triiodothyronine (T3) and serum thyroxine (T4) levels within the reference ranges (data not shown). Overall, 99 patients (80.49%) received levothyroxine, 17 (13.82%) were cured with methimazole, 3 (2.44%) took iodine and selenium supplements. Furthermore, 20 patients (16.26%) had a history of surgical thyroidectomy. Figure 1 shows the distribution of TDs in the cohort considered. Hashimoto’s thyroiditis and Graves’ disease were the most frequent pathologies of thyroid gland (51.22% and 13.82% of all TDs, respectively). Demographic and clinical characteristics of patients with TDs (classified in hypothyroidisms, hyperthyroidisms and TCs) are shown in Table 2. In this analysis we did not include 10/123 patients who suffered from an euthyroid goiter.

**Table 1:** Characteristics of the study population.

	NTD n = 6220	TD n = 123	Total n = 6343	p
Age at the end of follow up, mean years (SD) Male gender, n (%) HCV Ab positive, n (%) HBsAg positive, n (%) AIDS, n (%) Last T CD4+ ≥200/mm^3^, n (%) Last viral load ≥50 cp/ml, n (%) Age at TD diagnosis, mean years (SD) T CD4+ at TD diagnosis ≥200/mm^3^, n (%)	47.30 (10.98)	47.15 (11.56)	47.3 (11.9)	0.84
	4483 (72.07)	64 (52.03)	4547 (71.68)	<0.001
	2429 (39.05)	50 (40.65)	2479 (39.08)	0.81
	528 (8.49)	14 (11.38)	542 (8.54)	0.33
	4549 (73.13)	88 (71.54)	4637 (73.10)	0.70
	5934 (95.40)	111 (90.24)	6045 (95.30)	0.12
	419 (6.74)	6 (4.88)	425 (6.70)	<0.001
	NA	46.6 (11.5)	NA	
	NA	107 (86.99)	NA	

TD: thyroid disease; NTD: no thyroid disease; SD: standard deviation; NA: not applicable.

**Table 2:** Demographic, clinical and immunological characteristics of patients with symptomatic thyroid diseases (n = 113), classified in three groups as follows: hypothyroidisms, hyperthyroidisms and primitive thyroid cancers.

	Hypothyroidism n = 81	Hyperthyroidism n = 21	Primitive Thyroid Cancers n = 11
Male gender, n (%)	42 (51.85)	11 (52.38)	6 (54.54)
Age at TD diagnosis, mean years (SD)	45.80 (11.49)	42.52 (12.58)	48.64 (6.30)
HCV Ab positive, n (%)	30 (37.04)	9 (42.86)	4 (36.36)
HBsAg positive, n (%)	4 (4.94)	3 (14.29)	2 (18.18)
Delay from HIV diagnosis to TD onset, years (SD)	11.23 (10.20)	12.06 (8.14)	8.69 (9.88)
Time from cART start to TD onset, years (SD)	6.03 (9.07)	7.96 (6.12)	5.67 (5.85)
CD4+ T cell nadir/mm^3^, n (SD)	199.54 (162.34)	105.74 (169.30)	174.36 (106.34)
CD4+/CD8+ (SD)	0.33 (0.40)	0.29 (0.31)	0.17 (0.14)
CD4+ T cell increase from nadir to TD diagnosis, n (SD)	288.89 (255.77)	437.54 (306.13)	316.20 (304.01)
AIDS, n (%)	22 (27.16)	11 (52.38)	2 (18.18)
Comorbities ≥ 1, n (%)	60 (74.07)	16 (76.19)	10 (90.91)
Hypertension	27 (33.33)	9 (42.86)	5 (45.45)
Osteopenia/osteoporosis	18 (22.22)	3 (14.29)	3 (27.27)
Diabetes mellitus	15 (18.52)	4 (19.05)	2 (18.18)
cART at TD diagnosis			
INI + NRTI, n (%)	2 (2.47)	6 (28.57)	2 (18.18)
PI + NRTI, n (%)	69 (85.19)	12 (57.14)	1 (9.09)
NNRTI + NRTI, n (%)	6 (7.41)	2 (9.52)	3 (27.27)
Other, n (%)	4 (4.94)	1 (4.76)	5 (45.45)

SD: standard deviation; TD: thyroid disease; cART: combined antiretroviral therapy; INI: integrase inhibitor; NRTI: nucleoside reverse transcriptase inhibitor; PI: protease inhibitor; NNRTI: non-nucleoside reverse transcriptase inhibitor.

### Patients with hypothyroidism (n = 81)

Hypothyroidism was the most prevalent TD in our cohort (81 out of 113, 71.68%); the majority of patients (n = 63, 77.78%) had a Hashimoto’s thyroiditis. Mean age was 45.80 ± 11.49 years; slightly more than half was represented by males (n = 42, 51.85%) and overall 74.07% of subjects presented at least one comorbidity. Concerning HIV status, 22 patients (27.16%) experienced a previous AIDS-defining event, with a mean time from HIV acquisition to TD diagnosis of 11.23 years (SD: 10.20). At TD diagnosis all patients with hypothyroidism were on stable cART, the majority with a PI-containing regimen (n = 69, 85.19%). At the end of follow-up, all patients with hypothyroidism received a levothyroxine-based treatment. At the multivariate regression logistic analysis, hypothyroidism was negatively associated with male gender (OR = 0.41, 95% CI 0.26–0.65; p < 0.001) while age at HIV test per 10-year increase was borderline positively associated (OR = 1.21, 95% CI 1.00–1.46; p = 0.05).

### Patients with hyperthyroidism (n = 21)

The 18.5% (21/113) of patients with TD showed a symptomatic hyperthyroidism; the majority of them (n = 17, 80.95%) suffered from Graves’ disease. Males represented 52.38% of this group (n = 11); mean age was 42.52 years (SD: 12.58). 76.19% of patients presented at least one comorbidity. Mean time from HIV to TD diagnosis was 12.06 years (SD: 8.14), mean T CD4+ nadir was 105.74 cell/mm^3^ (SD: 169.30). At TD diagnosis the totality of patients with hyperthyroidism received cART, 57.14% (n =  512) with a PI-based regimen. At the end of study, all patients were treated for their thyroid dysfunction (17 with methimazole, 4 with levothyroxine), while 5 underwent thyroidectomy. At the multivariate regression logistic analysis, only T CD4+ cell nadir <200 cell/mm^3^ was independently associated with hyperthyroidism symptomatic disorders (OR = 3.08, 95% CI 1.06–1.45; p = 0.005).

### Patients with primitive thyroid cancer (n = 11)

Eleven subjects were diagnosed with TC during the study period. Papillary thyroid cancer was the most frequent TC (n = 7, 63.63%), followed by medullary (n = 2, 18.18%) and follicular thyroid cancer (n = 1, 9.1%). The histotype was not recorded for one patient. In this group, mean age was 48.64 years (SD: 6.30) and patients were equally divided by gender (45.5% females). Almost all subjects (n = 10, 90.91%) presented at least one comorbidity (cardiovascular, diabetes or osteopenia), while 36.36% (n = 4) were coinfected with HCV. TC was meanly diagnosed after 8.69 years from HIV diagnosis (SD: 9.88) and 100% of patients was on stable cART at that time (63.64% showing an undetectable viremia, with a mean T CD4+ cell count of 527 cell/mm^3^). All subjects underwent thyroidectomy and all received a substitution therapy with sodic levothyroxine. One of the patients with papillary thyroid cancer had a metastatic disease involving lungs and bones; he finally died, after six years of chemotherapies and radiotherapies. TC diagnosis was made at 47 years old; patient remained in good viro-immunological conditions until the exitus (at the last exams available: HIVRNA not detectable, T CD4+ 510 cell/mm^3^). To date, the rest of patients diagnosed with TC are in good conditions. At the multivariate logistic regression analysis TC diagnosis was independently associated only with age at HIV test per 10-year old increase (OR = 1.68, 95% CI 1.05‐2.66; p = 0.03).

### Incidence of thyroid diseases from 2005 to 2016 in our cohort

For the incidence analysis we limited the period from 2005 to 2016. We included 5337 patients with at least 2 available visits during this period, excluding prevalent cases (42251.71 person‐years of follow‐up). We observed 96 TDs incident cases with an overall crude incidence rate of 2.2 per 1000 persons (95% CI 1.8–2.7); the crude incidence rate was 1.6 per 1000 persons (95% CI 1.2–2.2) for males and 3.8 per 1000 persons (95% CI 2.9–5.1) for females. Demographics characteristics and TDs incidence rates for the entire follow-up considered (2005–2016) and for each 3-year period are shown in Table 3.

**Table 3:** Thyroid diseases incidence rates for the entire follow-up considered (2005–2016) and for each 3-year period.

Years	Gender	Total subjects (n, %)	Mean age (±SD)	TD diagnosis (n)	Person-years of follow-up	Incidence rate (95% CI)
2005–2016	F	1502 (28.14)	41.15 (10.37)	46	11865.62	3.88 (2.91–5.18)
	M	3835 (71.86)	44.49 (9.69)	50	30386.1	1.64 (1.24–2.17)
2005–2007	F	1086 (27.97)	38.88 (9.03)	6	2832.48	2.12 (0.95–4.72)
	M	2798 (72.04)	41.89 (8.43)	9	7228.32	1.25 (0.65–2.39)
2008–2010	F	1128 (28.23)	41.12 (9.63)	8	2986.03	2.68 (1.34–5.36)
	M	2868 (71.77)	44.30 (8.81)	10	7506.28	1.33 (0.72–2.48)
2011–2013	F	1271 (28.40)	42.63 (10.52)	17	3045.55	5.58 (3.47–8.98)
	M	3204 (71.60)	45.63 (9.75)	17	7768.06	2.19 (1.36–3.52)
2014–2016	F	1126 (27.85)	45.80 (10.49)	15	2993.36	5.01 (3.02–8.31)
	M	2917 (72.15)	48.56 (9.78)	14	7862.49	1.78 (1.05–3.01)

Temporal trend was also analyzed by Poisson regression as incident relative risk (IRR). For each 3-year period TDs incidence increased of 25%, with an IRR of 1.25 (95% CI 1.04–1.50; p = 0.02), mainly attributed to an incidence increase in females (IRR = 1.16, 95%CI 0.90-1.49; p = 0.24  for males and IRR = 1.36, 95%CI 1.03-1.78; p = 0.03  for females).

As expected, at the sensitivity analyses excluding subjects with positive HCV antibodies and with an AIDS-defining diagnosis we found only few differences for both the incidence and the prevalence of TDs due to the small number of total patients with symptomatic thyroid disorders considered (data not shown).

## Discussion

Here we report that TDs, including cancers, are a rare event in PLWH, with double incidence for females compared to males. Thus, in our cohort, only 1.94% of HIV-infected patients were diagnosed with thyroid disturbances during the follow-up period: 1.28% showed symptomatic hypothyroidism, 0.33% symptomatic hyperthyroidism, while thyroid cancer was documented in 0.17% of the considered population.

Other studies reported a significative higher prevalence of TDs in PLWH, ranging from 16% to 33.1%^11,26–29^, since they included also subclinical TDs in their analysis. If we compare the sole prevalence of overt hypo and hyperthyroidism in HIV-positive individuals, our data are similar to those found by other Authors, although their studies were performed in much smaller cohorts^17,30–32^. Moreover, in our cohort all patients were on stable cART at TD diagnosis, confirming that well treated PLWH are not at an increased risk of thyroid dysfunction, as previous described^14–17^.

However, it is not easy to make comparisons among distinct epidemiologic studies, due to the diversity of populations and end-points analyzed. Differences involve disease definition and severity (e.g. overt vs subclinical dysfunction), selection criteria, different reference ranges and laboratory techniques used to measure serum thyroid hormone levels^33^, besides the influence of age, sex and environmental factors^34^. For these reasons and considering the high prevalence of subclinical thyroid dysfunctions in HIV-infected subjects, we analyzed only symptomatic diseases.

The two major autoimmune TDs, Hashimoto’s thyroiditis and Graves’ disease, were the most frequent pathologies of thyroid gland in our cohort. Although small researches reported a prevalence of Hashimoto thyroiditis up to 2.6% in HIV-positive patients^35^, a large-population based study^36^ evaluated the presence of autoimmune diseases among 5186 HIV-infected patients, finding only 1 case of Hashimoto’s thyroiditis and 2 cases of Graves’ disease. Thus, these TDs seem to be more frequent in our cohort, showing a prevalence comparable to international general population values^34^.

Autoimmune TDs are much common among women than in men, with a female to male ratio ranging from 5:1 to 10:1 in general population^34^. The biological explanation for this gender difference is not entirely clarified, but the X chromosome inactivation could significantly contribute to the high incidence of autoimmune TDs in females^37^; moreover, it seems that maternal immune responses against fetal antigens can trigger autoimmune processes^38^. In our cohort of PLWH, male gender was confirmed as a protective factor for TDs onset, especially for hypothyroidism.

TDs development is also influenced by ageing in general population^39^. In our study, age at HIV test emerged as a variable associated with both hypothyroidism and TCs. Notably, we also focused on temporal trends, finding an increase in TDs incidence for each 3-year period from 2005 to 2016, attributable to the ageing of our cohort. Thus, increasing age and non-AIDS related comorbidities (which were highly represented in our cohort, with a peak of 90.91% of prevalence for TCs), together with chronic inflammation, immunosenescence and polypharmacy may play a role in TDs occurrence and progression.

However, immune restoration is also probably implicated in TDs development. As a matter of fact, we found that a T CD4+ cell nadir <200 cell/mm^3^ was independently associated with symptomatic hyperthyroidism. In this group we reported the most remarkable CD4+ T cell increase from nadir to TD diagnosis (mean CD4+ increase: 437.54 ± 306.13), supporting the previously described hypothesis of Graves’ disease as a manifestation of delayed immune reconstitution inflammatory syndrome (IRIS)^21,22^ and suggesting that some CD4+ T lymphocytes subsets may affect its occurrence^40^.

Finally, we did not find any correlation between HCV coinfection and thyroid abnormalities, although some Authors highlighted the role of both HBV and HCV in increasing the probability of thyroid dysfunction, especially hypothyroidism^20,29^.

In the post-HAART period, PLWH reported an increasing risk of non-AIDS-defining cancers that it is higher compared to uninfected controls^41–43^. To our knowledge, our study is the first one to evaluate TCs prevalence and associated risk factors in a population of HIV-infected patients. According to the latest Italian general population tumor report^44^, the risk to develop a TC is much higher in females compared to males; furthermore, although TC incidence is increasing, the overall mortality remains extremely low. In our study, TC seems to be a very rare condition, with a lower prevalence than the one reported for general population (0.17 vs 0.33%, respectively). In this population of PLWH, patients diagnosed with TC were equally divided by gender(although females represent less than 30% of the total cohort) and age was the only risk factor associated with thyroid tumor development. Italian data^44^ show that, from 2010 to 2014, 82% of TCs was represented by papillary thyroid cancer, followed by follicular (7%), medullary (4%) and anaplastic (1%) histotypes. As expected, in our cohort of PLWH, the most frequent TC was represented by papillary thyroid cancer. Interestingly, medullary thyroid cancer was diagnosed in almost 20% of patients considered in our study, with a percentage significantly higher compared to the Italian general population. Although medullary thyroid cancer development was associated with mutations of RET tyrosine kinase, no information is available about a possible interaction between HIV and thyroid tissue, as described for EBV^45^. However, due to our limited sample size, more researches are needed in order to confirm our results and to evaluate the real distribution of TCs in a population of HIV-infected individuals. A recent study^46^, performed using data from  > 6 million US patients from the National Cancer Data Base (NCDB), showed that PLWH were more likely to be diagnosed with advanced-stage of TCs and to experience a higher mortality after TC diagnosis compared to general population. At TC diagnosis, all individuals considered in our cohort were on stable cART, the majority with a satisfying viro-immunological profile; moreover, at the end of follow-up, TC was controlled in all patients except one, who died.

Our study should be interpreted within its limitations, firstly the retrospective monocentric design, which does not permit a more comprehensive description of HIV and immune system dynamicity. Secondly, although every TD diagnosis was accurately verified, there could be some bias in estimating the prevalence of thyroid disturbances in the population considered. Finally, all participants are from the same geographical area and it is reasonable to assume a similar iodine intake; however, our results may not apply to regions with different iodine consumption.

The strengths of this study include the large population size, the enrolment of an unselected group of HIV-infected patients and an accurate retrieval of data on TDs thanks to the use of two different sources, the clinic database and the HPADB of Brescia Province.

## Conclusions

To conclude, our study is the first to analyze the prevalence of exclusively symptomatic TDs (with accurate retrieval of diagnosis) in a large cohort of HIV-infected patients, confirming age and gender as crucial elements in the onset of thyroid abnormalities and highlighting the role of T CD4+ cell nadir as additional factor. Despite the high prevalence of thyroid dysfunction described in PLWH, symptomatic TDs remain a rare event. However, in the next decades, we will probably face a progressive increase of this comorbidity, considering that TDs (especially hypothyroidism) are dramatically more prevalent after the age of 40^47^ and that PLWH are actually ageing. Periodic screening with measurement of TSH levels could be implemented, in order to provide a rapid treatment and to minimize thyroid-related complications.

Moreover, we found an interesting much more elevated rate of medullary thyroid cancer in PLWH compared to the general population; however, the distribution of different histotypes and their extensive characterizations are yet to be fully described in this category of patients.
